# Computer‐assisted surgical planning and intraoperative guidance in fetal surgery: a systematic review†

**DOI:** 10.1002/pd.4660

**Published:** 2015-09-16

**Authors:** Rosalind Pratt, Jan Deprest, Tom Vercauteren, Sebastien Ourselin, Anna L. David

**Affiliations:** ^1^ Translational Imaging Group, CMICUniversity College LondonLondonUK; ^2^ Institute for Women's HealthUniversity College LondonLondonUK; ^3^ Department of ObstetricsUniversity Hospitals KU LeuvenLeuvenBelgium

## Abstract

Fetal surgery has become a clinical reality, with interventions for twin‐to‐twin transfusion syndrome (TTTS) and spina bifida demonstrated to improve outcome. Fetal imaging is evolving, with the use of 3D ultrasound and fetal MRI becoming more common in clinical practise. Medical imaging analysis is also changing, with technology being developed to assist surgeons by creating 3D virtual models that improve understanding of complex anatomy, and prove powerful tools in surgical planning and intraoperative guidance.

We introduce the concept of computer‐assisted surgical planning, and present the results of a systematic review of image reconstruction for fetal surgical planning that identified six articles using such technology.

Indications from other specialities suggest a benefit of surgical planning and guidance to improve outcomes. There is therefore an urgent need to develop fetal‐specific technology in order to improve fetal surgical outcome. © 2015 The Authors. Prenatal Diagnosis published by John Wiley & Sons Ltd.

## Abstract 2

**What's already known about this topic?**

- Fetal surgery has now become a clinical reality, with interventions such as laser treatment for twin‐to‐twin transfusion syndrome (TTTS) and open fetal surgery for spina bifida demonstrated in randomised control trials to improve neonatal outcome
- Other specialities are increasingly utilising computer‐assisted surgical planning software, with evidence that this can improve outcome

**What does this study add?**

- We feel that there is an urgent need to develop fetal‐specific technology for surgical planning as it is likely to play an important role in improving outcomes from fetal surgery

## Introduction

Imaging of the fetus using ultrasound, and increasingly magnetic resonance imaging (MRI), provides ever‐increasing diagnostic and prognostic information, which guides parental counselling and consideration of and decision making about mode and time of delivery. Fetal surgery has now become a clinical reality,^1^ with interventions such as laser treatment for twin‐to‐twin transfusion syndrome (TTTS) and open fetal surgery for spina bifida demonstrated in randomised control trials to improve neonatal outcome.^2^, ^3^ Fetal interventions are likely to increase in breadth and prevalence as surgical techniques and technologies improve, and as more procedures are proven to be clinically effective.^4^ As in any surgery, the best outcomes are likely when surgeons are prepared preoperatively with an in‐depth understanding of the anatomy that they will confront. In other surgical specialties medical imaging can now provide patient‐specific virtual 3D models so that surgeons can more easily understand a 3D map of the individual's anatomy before commencing surgery. 3D reconstruction can also be taken into the operating theatre for intra‐operative guidance.

In this review we first briefly present the processes involved in surgical planning and the technology being developed to manipulate fetal imaging and reconstruct 3D virtual fetal models for surgical planning. The results of a systematic review on the use of fetal image reconstruction to aid surgical planning are then presented. We finally consider the current state of surgical planning in other specialities, and how challenges faced in fetal surgery may benefit from surgical planning.

### Surgical planning

**Figure 1:**
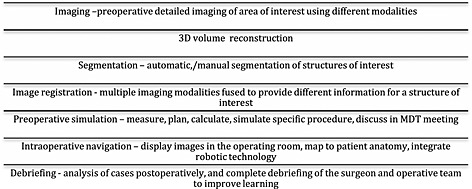
The processes involved in surgical planning^5^

For the purpose of this review surgical planning refers to computer‐assisted preoperative modelling and visualisation of anatomy in order to predefine the surgical steps, determine the best plan and transfer it to reality for an individual patient. Figure 1, 5 demonstrates the potential processes involved in surgical planning, although the precise steps involved will depend on the specific nature of the surgical intervention. Surgical specialties such as cranial, orthopaedic, hepatic and ear, nose and throat surgery are increasingly using these techniques for complex procedures.

The first stage in surgical planning is to take high quality images of the structures of interest. The images are then manipulated using specifically developed software to perform surface and volume rendering. Different imaging modalities can be fused to gain additional anatomical information.^5^, ^6^ Segmentation, the process of delineating different structures of interest, is performed, and colour can be applied, producing a virtual 3D model demonstrating structural relationships. The resulting model allows the surgeon to view the anatomy from different angles, and to understand the complex anatomical relationships of different structures prior to surgery, improving their understanding and development of a mental map of the patients' specific anatomy. In addition the software can aid surgeons in planning. Similarly to GPS navigation systems, that given constraints such as starting and finishing points and areas to avoid will calculate the best possible route, surgical navigation software can perform complex calculations, such as best points of entry to reach an area of interest at a given angle, avoiding other key structures.^7^, ^8^, ^9^ Software allows the surgeon, their theatre team and colleagues to ‘walk through’ the operation prior to performing the surgery. The imaging can also be shown to the patient to improve their understanding of their illness, the process of the operation, and the possible complications that may occur, which assists the attainment of informed consent.^10^

Finally the 3D model can be used in association with instrument tracking systems to provide guidance intra‐operatively. Augmented reality aligns preoperative spatially accurate segmented medical imaging over real‐time laparoscopic views, or imaging can be visualised in parallel on additional screens. This demonstrates the position of surgical instrument in relation to sub‐surface structures to the surgeon, and helps align and guide them into the optimal position reaching the area of interest in the best possible orientation, and thus improving outcome and reducing complication rate.^6^, ^11^, ^12^, ^13^, ^14^, ^15^

### Fetal imaging

3D and 4D ultrasound is now becoming more widely available within the clinical setting, and has notably been found to be useful in the diagnosis of facial, neural tube and skeletal abnormalities.^16^, ^17^, ^18^ However there is no example of 3D ultrasound imaging being used in computer‐assisted fetal surgical planning, MRI being the preferred imaging modality. Ultrasound is used for intra‐operative guidance in ex utero intrapartum treatment (EXIT) procedures to plan optimal site for hysterotomy, by mapping the placental location and fetal position.^19^ Ultrasound is also used in surgical intervention to guide needle placement because of its real‐time spatiotemporal resolution. Ultrasound is therefore of particular interest in surgical guidance.

Magnetic resonance imaging (MRI) is being increasingly employed as an adjunct to ultrasound imaging, and is generally accepted to be safe.^20^, ^21^, ^22^, ^23^, ^24^, ^25^ MRI can overcome some of the limitations of ultrasound, such as maternal obesity, oligohydramnios and fetal positioning^26^ to further investigate anatomical abnormality not clearly visualised on ultrasound. It also offers improved soft tissue contrast making it particularly helpful for imaging of the central nervous system (CNS), lungs, kidneys and placenta, and offers a larger field of view.

As the fetus is not sedated, a major difficulty with fetal MRI is movement artefact. For this reason instead of 3D volumetric imaging, which takes time to acquire, snapshot imaging techniques are utilised, such as half‐Fourier turbo spin echo (HASTE) and single shot fast spin echo (SSFSE). These produce a stack of motion‐frozen 2D slices with an in‐plane spatial resolution of around 1 mm, allowing clinical interpretation.^27^

### 3D reconstruction and segmentation of fetal MRI

For imaging to be useful for computer‐assisted surgical planning 3D volumes are required. Reconstructing 3D fetal images from 2D MRI slices is challenging. Difficulties include low image resolution, low signal‐to‐noise contrast, maternal and fetal movement that is unpredictable and can be large and the rapid development of fetal organs with gestation causing the shape and size of the structure of interest to change significantly.^28^

In order to create a 3D model from 2D slices fetal movement needs to be corrected for. Slices are usually acquired in an interleaved manner so as to reduce scan time whilst avoiding slice cross‐talk artefacts.^26^, ^29^ To reconstruct the resulting mutually inconsistent freeze frozen stack of 2D images to a volumetric 3D image of accuracy suitable for clinical use, the 2D slices need to be aligned to correct for movement, and combined to form precise volumes.

Various approaches have been developed to tackle the difficulties of autocorrecting motion corrupted slices, as summarised in Table 1. Automatic realignment involves reconstruction–alignment methodology that forms a 3D volume from scattered slices and then refines the alignment in an iterative framework.^30^, ^31^, ^32^, ^33^ The best results are possible when several repeats of all orthogonal planes are used, providing repeat information in all three planar views.^30^, ^34^, ^35^, ^36^

**Table 1:**
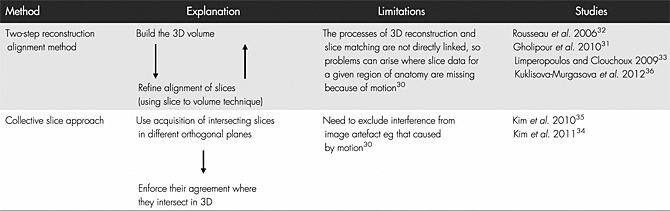
Techniques developed to overcome fetal motion and create 3D volumetric images from a stack of 2D motion frozen MRI images

Prior to reconstruction, the tissue of interest needs to be identified, excluding all other fetal and maternal tissue information that would interfere with the algorithms used in 3D reconstruction. For clinical application, this identification, a form of segmentation, needs to be automatic, or semi‐automatic. Ideally the ‘recognition’ of fetal structures should involve minimal user input, reducing the time and financial costs for clinicians, and also preventing inter‐observer variability and therefore standardising the results.

Many automated segmentation techniques focus on the process of comparing images to a pre‐defined gestation specific reference atlas to identify structures of interest.^37^, ^38^, ^39^, ^40^ Alternative semi‐automatic statistical approaches are being considered that use machine learning methods to segment structures of interest without the need of a reference atlas,^41^, ^42^, ^43^ avoiding the difficulty associated with changes in structure with increasing gestational age.

Most 3D reconstruction and segmentation work has been performed on fetal brain, a relatively rigid structure with minimal motion outside that of head movement. However, the techniques developed could be translated to reconstruct and segment other fetal structures, and have been used to reconstruct the fetal thorax and perform automated lung segmentation, demonstrating accurate fetal lung volume calculation from 20 to 38 week gestation.^44^ The goal of 3D reconstruction of moving organs, for example the bowel, is even more complex and requires further technological development.

We are interested in how this developing field of technology can be utilised in relation to fetal surgery, with a goal of improving outcome.

## Method

We performed a systematic review on the use of image reconstruction in fetal surgical planning to see how surgical planning tools have been employed in clinical practise.

To search the literature MeSH terms and keywords relating to the image reconstruction (3D reconstruction, 3D model, virtual reality, augmented reality, computer assisted surgery, 3D imaging and computer simulation) were combined with terms for fetal surgery (fetal/foetal surgery and fetal/foetal surgical planning). PUBMED and EMBASE were searched electronically on 13 April 2015. Reference lists of relevant articles and reviews were hand searched by RP for additional literature, and the articles that cited them reviewed. Titles and abstracts were screened for relevance by RP. Full text copies of relevant articles were retrieved and read in full by RP who agreed the final list with ALD.

Studies published at any time that discussed the use of image reconstruction specifically for surgical planning, and not for diagnostic or prognostic reasons alone, were included. Participants were pregnant women with normal or complicated single or multiple pregnancies. Animal studies were excluded.

## Results

A total of 248 studies were identified, of which 215 were excluded after review of the title and abstract. The remaining 33 were read in full, and 26 excluded for not relating to surgical planning, or not involving image reconstruction or 3D imaging. The six included studies describe cases in which surgical planning tools were used for treatment of fetal structural abnormality such as airway obstruction, facial cleft and spina bifida, or twin complications (Table 2). Each study reported on a few cases in which evidence of efficacy was based on subjective operator experience during the surgical procedure. No studies presented objective assessment of operating time, complication rate or outcome of surgery.

**Table 2:** Summary of the six included studies demonstrating computer‐assisted 3D reconstruction, and the impact of fetal surgical planning

Author	Summary	Method	Software	Impact
Werner *et al*. 2014^45^ ^n}^	Printed 3D models demonstrate complex fetal anatomy	3D virtual models created from MRI and CT demonstrating cervical lymphangioma, spina bifida, cleft lip	Mimics, Materialise, Leuven, Belgium. Autodesk Mudbox, San Francisco,California	None demonstrated with regards planning
Werner *et al*. 2015^46^ ^n}^	3D model of a fetus with lumbosacral myelomeningocele	3D virtual models created from MRI	Mimics, Materialise, Leuven, Belgium	None demonstrated with regards surgical planning
Norwitz *et al*. 2000^47^ ^n}^	Preoperative surgical planning for conjoined twins with twin reversed‐arterial‐perfusion sequence requiring immediate separation after delivery	3D virtual model constructed from MRI demonstrated the joined liver and biliary tree anatomy	Slicer, Surgical planning Laboratory of Brigham and Women's Hospital, Boston	Increased preoperative anatomical understanding
Werner *et al*. 2011^48^ ^n}^	3D model to demonstrate virtual bronchoscopy, on a normal fetus	3D model of fetal airway from which a simulation bronchoscopy movie was created, demonstrating the fetal airway patency	Mimics, Materialise, Leuven, Belgium	Virtual bronchoscopy allowed confident assessment of fetal airway to plan delivery
Werner *et al*. 2013^49^ ^n}^	3D models to assess airway patency in four fetuses with complex neck masses	3D model of fetal airway from which a simulation bronchoscopy movie was created, demonstrating the fetal airway patency	Mimics, Materialise, Leuven, Belgium	Virtual bronchoscopy allowed confident assessment of fetal airway to plan delivery
Luks *et al*. 2001^50^ ^n}^	Preoperative planning for laser treatment of twin‐to‐twin transfusion syndrome	Virtual reconstruction used to study location of placental umbilical cord insertion and inter‐twin membrane, to calculate optimum port entry point, and the length and angle of curved instrument required to reach the target area	SNN 3.0 Planning and Navigation Software, Surgical Navigation Network, Mississauga, Ontario, Canada	Improved preoperative anatomical understanding

All included studies used commercially available 3D image viewing software designed for other specialities, and manual segmentation. They are therefore limited in their anatomical accuracy, as commercial 3D reconstruction software has not yet been optimised for fetal imaging, as discussed above. They are also limited by the time constraints of manual segmentation.

3D reconstruction has been performed on fetuses with multiple abnormalities in order to demonstrate complex anatomy prior to surgery.^45^, ^46^ Cervical lymphangiomas,^45^ spina bifida^46^ and cleft lips^45^ have all been reconstructed. Although this added little to the clinical management of these cases, it proves that the concept is possible. The complex anatomy of conjoined twins has also been reconstructed,^47^ and this has been shown to be useful in providing surgeons with improved anatomical understanding prior to surgery, in this case clearly demonstrating the shared liver but separate biliary trees.

Two groups have gone further in using computer reconstructions to assist management decisions. Werner *et al*.^48^, ^49^ made 3D reconstructions of four fetuses with complex cervical tumours (three lymphangiomas and one teratoma) from which virtual bronchoscopy videos were created demonstrating airway patency (Video available at: http://onlinelibrary.wiley.com/doi/10.1002/uog.11162/full). Virtual bronchoscopy was successful in all cases, demonstrating no tracheal invasion and patent airways in all cases. EXIT procedure was therefore avoided, and all babies were delivered by caesarean section with good postnatal outcome.

Luks *et al*.^50^ created two 3D reconstructions of monochorionic twins with TTTS to plan fetoscopic laser ablation of communicating placental vessels. 3D reconstructions were used to study the anatomy of each amniotic cavity, the point of placental insertion of the umbilical cord and the location of the inter‐twin membrane in relation to the proposed port placement. Three models were used to calculate best point of port entry, and the length and angle of curved instrument required to reach the target area at the desired angle. The 3D reconstruction was reported to improve anatomical understanding preoperatively, and allowed manipulation of the imaging to facilitate surgical planning, therefore demonstrating successful implementation of a 3D reconstruction for surgical planning.

## Discussion

This systematic review of fetal surgical planning identified six relevant studies in which surgical planning tools were used for treatment of fetal structural abnormality or twin complications. Subjectively the application of medical image computing improved pre‐operative planning, but it was not possible to identify if there was an impact on fetal, maternal or neonatal outcome, because of the small number of studies.

Minimally invasive endoscopic abdominal surgery is a broadly similar surgical technique to fetoscopic surgery, in which surgical planning software is being increasingly investigated in laparoscopic renal, liver and pancreatic surgery, with some evidence of improved outcome. Preoperative planning and real‐time assisted navigation using 3D reconstructions in laparoscopic partial nephrectomy have been shown to reduce operation time and blood loss when compared to controls,^12^ and 3D reconstruction for surgical planning shortened operating time and reduced hepatic inflow occlusion in surgical treatment of centrally located hepatocellular carcinomas.^13^

Surgical planning and intraoperative guidance are also used extensively in cranial surgery, ear, nose and throat surgery, and orthopaedics. Examples of different computer‐assisted planning and intraoperative tools available commercially in different specialities are given in Table 3. Software is designed to use Digital Image and Communications in Medicine standard (DICOM) files, with some systems linking seamlessly with a Picture Archiving and Communication System (PACS). They are intuitive to use, requiring minimal training and can facilitate discussion in multidisciplinary team (MDT) meetings and in patient education and consent. As well as producing 3D virtual models with segmentation, the software provides multiple tools that can merge different imaging modalities, such as ultrasound, CT and MRI, and anatomical with functional imaging, to give maximum information on one model. These models can be used to compute the best port entry site to reach the target area of interest whilst avoiding other key structures, or calculating the implants needed for orthopaedic surgery.^11^, ^51^ Models can also be manipulated to show virtual endoscopic views, for example of the sinuses, bowel or respiratory tract.^52^, ^53^, ^54^ Models can be taken into the operating theatre and fused with intraoperative image such as real‐time ultrasound, CT and MRI.^55^, ^56^ Finally they can be used with instrument tracking technology such as optical surgical navigation cameras or electromagnetic systems to provide intraoperative real‐time guidance of instruments on preoperative/intraoperative imaging.^10^, ^11^

**Table 3:** Computer‐assisted surgical planning and intraoperative guidance tools available commercially in different specialities

Speciality	Companies developing software	Available features
Cranial surgery	Brainlab, Fujifilm Medical Systems USA, GE Healthcare, Medtronics, Micromar, Renishaw, Scopis Medical, SonoWand, Stryker, Synaptive	• Data fusion of CT and MRI, and functional and anatomical datasets
		• Demonstrate cortical surface and vasculature anatomy
		• Automatically identify whether a trajectory is suitable for the current configuration and so plan the optimal approach
		• Place targets and trajectories together with a safety zone to determine if a trajectory passes too close to key anatomy
		• Plan the extent of tumour resection
		• Craniotomy simulation
		• Intraoperative imaging integration—intraoperative CT, MRI and USS fusion
		• Integrated tracking technologies
Ear, nose and throat surgery	Brainlab, ClaroNav, Fiagon Dynamic Navigation, GE Healthcare, Medtronics, Scopis Medical, Stryker	• Unique perspective views of patients 3D anatomy including virtual endoscopy
		• Advanced automated segmentation for tumours, skin, brain, vasculature and ventricle
		• Automated image fusion between CT, MRI, CTA, MRA, fMRI and PET
Orthopaedic surgery	BlueBelt Technologies, Brainlab, Materialise, Medacta, Medtronics, OrthAlign, Stryker, VoyantHealth	• Contralateral, healthy side can be mirrored, precisely aligned and used as a template of normality
		• Analyse mechanical axial axes in 3D
		• Plan osteotomy, and plate and screw placement in order to accurately restore anatomy
		• Cutting guides can be designed and manufactured through 3D printing to guide saw blades, and drilling guides to guide placement and angle of screw insertion
		• Plan plates, screws or hips needed for any given procedure with high accuracy so ready in operating theatre
		• Advanced templating functions can be used to plan deformity corrections including external fixation, and paediatric measurements including growth calculators
		• Intraoperative mapping of anatomy
Gastrointestinal surgery—renal, hepatic, pancreatic and bowel	EDDA technology, CasCination, Fujifilm Medical Systems USA, Intrasense, Pathfinder	• 3D visualisation of pathology and surrounding anatomy such as feeding blood vessels
		• Plan optimal path of therapeutic delivery and transection plane in 3D
		• Patient specific surgical simulation
		• Automatic calculations of resected and residual tissue volumes
		• Calculation of vascular territories
		• Virtual endoscopy
		• Real‐time tracking and guidance of surgical instrument position relative to preoperative imaging and 3D models
		• Integration of real‐time intraoperative ultrasound imaging to preoperative imaging
Thoracic surgery	Fujifilm Medical Systems USA, Intrasense	• Quantitative evaluation and 3D visualisation of pathology, e.g. lung tumour
		• Calculation of the territories of pulmonary vessels and bronchi of the lung field region
		• Simulation of surgery and biopsy
		• Virtual brochoscopy

### The future of fetal surgical planning

In order to be of use for surgical planning 3D reconstruction needs to clearly demonstrate complex anatomy. Fetal MRI imaging is improving, with higher resolution and improved signal‐to‐noise ratio. The computer processing of this data to produce 3D reconstruction with segmentation is also developing, and so it seems timely that these technologies are integrated into a surgical planning platform. Such technology could help plan port placement for fetal and placental surgery and improve anatomical understanding through virtual bronchoscopy or cystoscopy.

Fusion of different imaging modalities, for example MRI for greater soft tissue anatomic detail with Ultrasound Doppler to demonstrate vasculature, could help further improve anatomical understanding. Fusion of fetoscopic images with preoperative imaging has already been developed, with work aimed at mosaicking endoscopic placental images onto 3D ultrasound placental images. This would allow surgeons performing laser treatment to visualise the entire placental anatomy, and not just the minimal field of view available through the scope.^57^, ^58^, ^59^, ^60^

Instrument tracking could also help surgeons perform more complex percutaneous procedures, demonstrating device position on 3D imaging and so helping to locate more precisely fetal target organs or vessels. As the boundaries of fetal intervention expand with new potential treatments, such as fetal pace makers^61^ and in‐utero stem cell transplantation and fetal gene therapy,^62^ developing guidance systems will ensure that therapeutic agents and devices can be accurately delivered to precise locations. Intraoperative guidance could also be combined with robotic technology, improving surgeon dexterity whilst reducing the invasiveness of procedures.

3D models may also have an important role in preoperative patient counselling, in order to improve the patients' understanding of the pathology, the planned operation, and to prepare patients for delivery of a baby with anatomical abnormalities. The increasing availability of high‐quality 3D printers at reasonable cost may further help prepare patients by providing models they can see, hold and even show to friends and family.

New technologies are also being investigated to assist surgical training, particularly in specialities where surgery is complex, and where the pathology is rare, reducing exposure within clinical practise. Efforts have been made to produce high‐fidelity simulators for fetal interventions.^63^ Their function may be improved by the ability to print anatomically accurate 3D models of different, complex clinical cases which could then be used to train and assess many surgeons in different geographical locations.^64^

## Conclusion

There is some early subjective evidence that 3D reconstruction of fetal imaging is beneficial for fetal surgical planning, but as of yet there are no studies on intraoperative guidance or studies using objective measures of outcome. Fetal surgery is increasingly being offered for the correction of structural malformations and newer fetal therapies are being considered for genetic disease. Indications from other surgical specialities suggest a benefit of computer‐assisted planning and guidance to improve outcomes. Given the constraints of operating in the intrauterine environment and the intricacy of the fetal anatomy, surgical planning tools are likely to play an important role in improving outcomes from fetal surgery, and there is therefore an urgent need to develop fetal‐specific technology for this purpose.

**WHAT'S ALREADY KNOWN ABOUT THIS TOPIC?**

- Fetal surgery has now become a clinical reality, with interventions such as laser treatment for twin‐to‐twin transfusion syndrome (TTTS) and open fetal surgery for spina bifida demonstrated in randomised control trials to improve neonatal outcome
- Other specialities are increasingly utilising computer‐assisted surgical planning software, with evidence that this can improve outcome

**WHAT DOES THIS STUDY ADD?**

- We feel that there is an urgent need to develop fetal‐specific technology for surgical planning as it is likely to play an important role in improving outcomes from fetal surgery
